# Prevalence and Associated Clinical Features of Type 1 Diabetes Mellitus Among Children Presented to a Tertiary Health Care Center of Himalayan Foothills

**DOI:** 10.7759/cureus.35435

**Published:** 2023-02-24

**Authors:** Henuka Verma, Prashant Kumar Verma, Vinod Kumar, Nowneet Bhat, Yogesh Bahurupi

**Affiliations:** 1 Pediatrics, All India Institute of Medical Sciences, Rishikesh, IND; 2 Community and Family Medicine, All India Institute of Medical Sciences, Rishikesh, IND

**Keywords:** diabetic complications, dibetes mellitus, prevalence, children, type 1 diabetes mellitus (t1d)

## Abstract

Introduction

Diabetes Mellitus (DM) is a complex metabolic disorder characterized by chronic hyperglycemia. Knowing its prevalence, associated clinical features, and complications is essential for diagnosing children having diabetes-like clinical features. Since there is a limited study from India and no similar study from this geographical part, the present study was carried out.

Material and method

It is a cross-sectional study, which includes children aged 1-18 years presented to the pediatric outpatient department (OPD), inpatient department (IPD), and emergency with clinical features of Type 1 Diabetes Mellitus (T1DM). The enrolled cases were assessed for confirmation of T1DM, and clinical features and associated complications were recorded in the case record form.

Result

A total of 218 children with clinical features of T1DM were enrolled, out of which 32 (14.7%) had T1DM. Among the 32 T1DM patients, 31 (96.9%) of the participants presented with polyuria, 29 (90.6%) had polydipsia, and 13 (40.6%) had polyphagia. Out of 32 children, 3 (9.38%) had diabetic neuropathy, and 1 (3.1%) had diabetic retinopathy.

Conclusion

We found that many children with diabetes have clinical features of T1DM and uncontrolled blood sugar. This emphasizes the need for early detection and treatment to prevent long-term complications.

## Introduction

Diabetes Mellitus (DM) is a complex metabolic disorder characterized by chronic hyperglycemia. The American Diabetes Association (ADA) classified diabetes into four major types: Type 2 diabetes, type 1 diabetes, and other specific types, including monogenic diabetes, drug or chemical-induced diseases of the exocrine pancreas, infection-induced, and gestational diabetes. The two most common forms of diabetes are Type 1 Diabetes Mellitus (T1DM), characterized primarily by deficiency of insulin secretion because of autoimmune pancreatic β-cell damage, and Type 2 Diabetes Mellitus (T2DM), which occurs due to insulin resistance along with β-cell impairment [1].

According to International Diabetes Federation Atlas 2019, the incidence of T1DM in children and adolescents aged less than 15 years is increasing in nearly all parts of the world, with an estimated increase of 3% per annum with geographic differences. India has the highest number of cases of T1DM in children and adolescents in terms of incidence and prevalence, with incident cases being 15900 per annum and estimated prevalent cases being 95600 per annum in 2019 [2].

The prevalence of T1DM has been studied by several methods; school records, national registries, hospital records, and surveys in different age groups in the developing world. The prevalence varies from as low as 0.09 per 1000 in China to as high as 3.40 in the United Kingdom [3]. "Incidence studies are available from many countries and reveal rates ranging from 36.5/100,000 in Finland and 36.6/100,000 in Sardinia (Italy) to 0.1-4.6/100,000 in China and 0.4/100,000 in Thailand" [4-6].

Studies have been done in different parts of India to determine the prevalence of T1DM and associated complications. However, those studies are primarily based in southern India and are very few. Since there are limited studies from India and no similar study was done from this geographical part, the present study was carried out.

## Materials and methods

It is a hospital-based observational study. The Institute Ethics Committee approved the study protocol (AIIMS/IEC/20/706). All children (1-18 years) presenting to AIIMS Rishikesh pediatric Inpatient Department (IPD), Outpatient Department (OPD), and Emergency department with clinical features of Type 1 Diabetes Mellitus were included in the study. The participants were provided with a participant information document; written consent was obtained and included in the study as per the inclusion and exclusion criteria.

Inclusion criteria included: Children of age 1 year to 18 years; Prior diagnosis of Type 1 Diabetes Mellitus; Children with clinical features suggestive of Type 1 Diabetes Mellitus

Exclusion criteria includes: Refusal of consent; Children <1 year of age; Other types of diabetes- Type 2 diabetes, Drug-induced, Pancreatic diabetes

Once children were included in the study, a baseline assessment was done, which included the following: Relevant history as per the predesigned proforma; Relevant Examination.

The cases were diagnosed as T1DM according to the International Society for Pediatric and Adolescent Diabetes (ISPAD) 2018 guidelines, clinical features of diabetes mellitus, and plasma glucose concentration ≥ 200 mg/dL. The socioeconomic status was categorized by using the updated version (2020) of the modified Kuppuswamy socioeconomic scale( based on occupation, education of family head, and family income); score ranges from < 5, 5-10, 11-15, 16-25, and 26-29 for lower, upper lower, lower middle, upper middle, and upper socioeconomic class, respectively. In confirmed cases, laboratory investigations were done for albumin creatinine ratio, lipid profile, free T4 (FT4) and Thyroid-stimulating hormone (TSH), tissue transglutaminase IgA (tTGA), HbA1c, and serum glutamic oxaloacetic transaminase/ serum glutamate pyruvate transaminase/ gamma-glutamyl transferase (SGOT/SGPT/GGT). The confirmed cases of T1DM were screened for associated complications; diabetic neuropathy, retinopathy, and nephropathy,

Statistical analysis

Data was entered using a Microsoft Excel spreadsheet and analyzed using professional statistics/SPSS version 23 for windows. Descriptive statistics were shown as means ± standard deviations for normal distribution values and medians/IQRs for continuous skewed distribution values and frequencies and percentages/proportions for categorical variables. Chi-square or Fisher exact test was used for categorical variables, and an independent t-test for numerical variables. A 5% probability (p-value less than 0.05) was considered statistically significant for all comparisons.

## Results

Two hundred eighteen children presented with clinical features of diabetes mellitus were included in the study over 18 months. Out of 218 study participants, 59.6% (130) were from Uttarakhand, 39.4% (86) of the participants belonged to Uttar Pradesh, and 0.5% were from Delhi and Haryana. Out of 218 participants, 14.7% (32) had T1DM, and 85.3% (186) of the study participants did not have confirmed T1DM. 58.3% (127) of the participants were males, and 41.7% (91) were females, with male to female ratio of 1.4:1. Among confirmed cases of T1DM, 17(53.1%) were male, and 15(46.9%)were female with male to female ratio of 1.13:1. Majority of the study participants (46.3%) belong to the lower middle class. Among confirmed T1DM cases, 18(56.2%) and 11(34.4%) cases belonged to Lower middle and upper lower socioeconomic status, respectively. Table 1 shows the baseline demographics of the study participants.

**Table 1:** Baseline Demographic profile of study participants ***Significant at p<0.05, 1: Wilcoxon-Mann-Whitney U Test, 2: Chi-Squared Test, 3: Fisher's Exact Test

Parameters	Type 1 Diabetes		p-value
	Yes (n = 32)	No (n = 186)	
Age (Years)	10.53 ± 4.50	8.99 ± 5.22	0.122^1^
Age			0.092^2^
1-5 Years	5 (15.6%)	61 (32.8%)	
5-12 Years	16 (50.0%)	62 (33.3%)	
12-18 Years	11 (34.4%)	63 (33.9%)	
Gender			0.524^2^
Male	17 (53.1%)	110 (59.1%)	
Female	15 (46.9%)	76 (40.9%)	
Socio-Economic Status			0.050^3^
Upper	1 (3.1%)	0 (0.0%)	
Upper Middle	2 (6.2%)	39 (21.0%)	
Lower Middle	18 (56.2%)	83 (44.6%)	
Upper Lower	11 (34.4%)	57 (30.6%)	
Lower	0 (0.0%)	7 (3.8%)	

**Figure 1:**
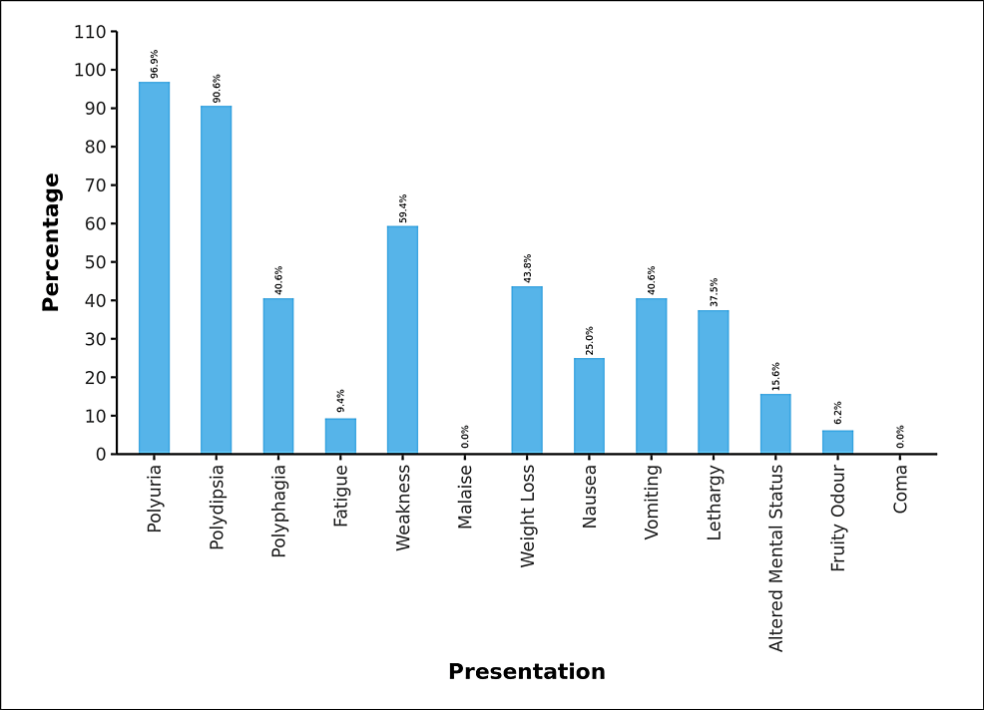
Summary of Clinical Presentation of children with type 1 Diabetes mellitus

Among the 32 T1DM patients, 31 (96.9%) of the participants presented with polyuria, 29 (90.6%) had polydipsia, and 13 (40.6%) had polyphagia. 3 (9.4%) of the participants had fatigue, and 19 (59.4%) patients presented with weakness. 14 (43.8%) participants complained of weight loss. 8 (25.0%) had nausea, and 13 (40.6%) had vomiting at presentation. 12 (37.5%) had lethargy, and 5 (15.6%) presented with altered mental status. 5 (15.6%) were presented with DKA. 2 (6.2%) of the participants had presented with a fruity odor. None of the patients presented with malaise or were in a coma, as shown in Figure 1.

The mean age of diagnosis among children with T1DM was 8.80 years. The Majority of the children had a high level of blood sugar at the time of enrolment in the study, with a median (IQR) value of 496 mg/dL (444.25-545). Of 32 cases with T1DM, 2 (7.7%) also took oral hypoglycemic agents. The mean HbA1C (%) value in children with T1DM was 13.5±2.8. Most children {n=20 (62.5%} had HbA1C between 10-15%. 3(9.4%) and 9(28.1%) children had HbA1C between 7-10% and >15%, respectively.

All 32 patients were screened for diabetic-associated complications; diabetic retinopathy and neuropathy were present in 1(3.1%) and 3(9.38%) cases, respectively. No children had features of diabetic nephropathy and macrovascular disease. The cases were screened for the associated autoimmune conditions; none had evidence of autoimmune hypothyroidism or autoimmune hepatitis. Out of 32 cases of T1DM, 2 (6.25%) had raised tTGA levels (>3 times the upper limit), although duodenal biopsy could not be done in those cases.

## Discussion

This study is a hospital-based observational study conducted in a tertiary health care center in Uttarakhand state of India. T1DM incidence is increasing in children and adolescents, as per the International Diabetes Federation Atlas 2019, in all parts of the world. The prevalence of T1DM in school children was 1.467% out of 92,047 in a survey conducted by the government of India under the National Program [7]. Studies have been done in different parts of India to determine the prevalence of T1DM and associated complications. However, those studies are mostly based in southern India and are very few. Hence, we assessed the prevalence of T1DM in our center. Out of 218 participants, 14.7% (32) of the participants had T1DM; a total of 16,344 children were presented to the Paediatrics department. This reveals that the hospital prevalence of T1DM is 1.95 per 1000 children. There are few studies and registries available in India which show the prevalence; the study done by Kalra S et al. in Haryana state reported a prevalence of 22.22/100,000 population (5-16 years) while in the 0-5 years age group prevalence is 3.82/100,000 [8]. Similarly, a study by Ramachandran A et al. in 1992 in South India found a prevalence of 0.26/1000 in children <15 years of age [9]. 

In our study among diabetic children (n=32), the mean age at the diagnosis was 8.8 ± 4.09 years. This is low compared to the finding by Praveen PA et al., with a mean age of diagnosis of 12.9 ± 6.5 years, and similar to a study done in Western India by Sharma B et al. with a mean age of 10.0 ± 3.63 years [10,11]. SEARCH registry of the US also shows lower mean age of diagnosis, i.e., 10 ± 4.5 years [12,13]. Our study's most common clinical presentation was osmotic symptoms (polyuria (96.9%) and polydipsia (90.6%)) followed by weakness (59.4%), weight loss (43.8%), polyphagia and vomiting (40.6%) both and lethargy (37.5%). Another less common presentation was nausea, altered mental status, fatigue, and fruity odor. In the study conducted by Praveen PA et al. at the Indian Council of Medical Research, the Majority of children presented with polyuria, polydipsia, and weight loss (28.8%) as compared to osmotic symptoms alone, which was (26.5%) [10]. This shows the importance of awareness about the common clinical features of T1DM among society and treating physicians for screening and early identification of T1DM.

Among diagnosed cases of T1DM, 53.1% and 46.9% were male and female, respectively. Other studies from India, like the YDR registry and the SEARCH registry from the US, show similar findings, with a male proportion of 52.9% and 53.3%, respectively [12,13].

On screening for associated complications, we found 1(3.1%) child had retinopathy, and 3 had neuropathy. No children had features of diabetic nephropathy and macrovascular disease. The mean (SD) LDL level was 94.38 (34.43) mg/dL, and 2 children had increased LDL levels of >100 mg/dL and were given dietary advice and repeat LDL levels on follow-up. In India, Sudhanshu S et al., in their study in Lucknow, found diabetic nephropathy in 3%, diabetic retinopathy in 3.6%, and raised LDL (>100 mg/dL) in 34% subjects out of 164 who were screened for complications [14]. Another study from South India by Ramachandran A et al. revealed nephropathy in 7.1%, sensory neuropathy in 3%, and 13.4 % of children with diabetic retinopathy [15].

All children were screened for autoimmune conditions associated with T1DM, i.e., autoimmune hypothyroidism, autoimmune hepatitis, and celiac disease. All the patients were screened negative for autoimmune hypothyroidism and hepatitis. 2 (6.25%) children had raised tTGA levels (>3 times the upper limit). The study by Sharma B et al., which enrolled 150 children, showed that 24.8% of children had celiac disease and 14.1% and 3.3% had hypothyroidism and Grave's disease, respectively [11]. The prevalence of T1DM-associated autoimmune conditions in the Indian context is still poorly studied.

In our study majority of children having T1DM belong to lower middle {n=18(56.2%)} and upper lower class {n=11 (34.4%)}. This is different from the Indian YDR and SEARCH registry, which found that 60.8% and 53.6% of children with T1DM are from high socioeconomic status, respectively. A possible explanation for this; is increased diagnosis among these children being frequently referred to our government institutions. The Diabetes Control and Complications Trial and its follow-up Epidemiology of Diabetes Interventions and Complications study reported that intensified insulin therapy, along with support and education, results in better long-term glucose control and delays the complications of T1DM [16-18].

The study's possible limitations are a small sample size and a hospital-based study. Since it is a time-bound study, no follow-up was done for subsequent associated complications.

## Conclusions

We found a significant number of children present with diabetes having clinical features like T1DM. It even involves lower and middle socioeconomic children, emphasizing the need for a surveillance system, early detection, and treatment to prevent long-term complications.
